# Development of a perfusion chamber assay to study in real time the kinetics of thrombosis and the antithrombotic characteristics of antiplatelet drugs

**DOI:** 10.1186/1477-9560-10-11

**Published:** 2012-08-01

**Authors:** Gillian Stephens, Ming He, Connie Wong, Marzena Jurek, Hans-Christian Luedemann, Golnaz Shapurian, Kevin Munnelly, Craig Muir, Pamela B Conley, David R Phillips, Patrick Andre

**Affiliations:** 1 Portola Pharmaceuticals Inc, 270 E. Grand Avenue, Ste 22, South San Francisco, CA 94080, USA; 2 Millennium Pharmaceuticals Inc, Cambridge, MA, USA

**Keywords:** Real time, Perfusion, Thrombosis, Kinetics, Antiplatelet drugs

## Abstract

**Background:**

Arterial thrombosis triggered by vascular injury is a balance between thrombus growth and thrombus fragmentation (dethrombosis). Unbalance towards thrombus growth can lead to vascular occlusion, downstream ischemia and tissue damage.

Here we describe the development of a simple methodology that allows for continuous real time monitoring and quantification of both processes during perfusion of human blood under arterial shear rate conditions. Using this methodology, we have studied the effects of antiplatelet agents targeting COX-1 (aspirin), P2Y_12_ (2-MeSAMP, clopidogrel), GP IIb-IIIa (eptifibatide) and their combinations on the kinetics of thrombosis over time.

**Results:**

Untreated samples of blood perfused over type III collagen at arterial rates of shear promoted the growth of stable thrombi. Modulation by eptifibatide affected thrombus growth, while that mediated by 2-MeSAMP and aspirin affected thrombus stability. Using this technique, we confirmed the primacy of continuous signaling by the ADP autocrine loop acting on P2Y_12_ in the maintenance of thrombus stability. Analysis of the kinetics of thrombosis revealed that continuous and prolonged analysis of thrombosis is required to capture the role of platelet signaling pathways in their entirety. Furthermore, studies evaluating the thrombotic profiles of 20 healthy volunteers treated with aspirin, clopidogrel or their combination indicated that while three individuals did not benefits from either aspirin or clopidogrel treatments, all individuals displayed marked destabilization profiles when treated with the combination regimen.

**Conclusions:**

These results show the utility of a simple perfusion chamber technology to assess in real time the activity of antiplatelet drugs and their combinations. It offers the opportunity to perform pharmacodynamic monitoring of arterial thrombosis in clinical trials and to investigate novel strategies directed at inhibiting thrombus stability in the management of cardiovascular disease.

## Background

It is recognized that formation of arterial thrombi (thrombus growth) depends on platelet activation, resulting in the activation of the receptor function of GP IIb-IIIa for fibrinogen and von Willebrand factor to mediate platelet aggregation. Under shear stress conditions such as those found in stenosed arteries, platelet activation triggered by either von Willebrand factor interactions with GP Ibα and GP IIb-IIIa [1-4], GP VI binding to collagen [5], or ADP and TxA_2_ binding to their respective receptors (P2Y_12_, TP) [6] have been shown to mediate thrombus growth. Unlike for the effectors of thrombus growth, our knowledge of the effectors of dethrombosis is limited, in part due to a lack of appropriate technology. It is known that fibrin stabilizes platelet-rich arterial thrombi (accounting for the ability of fibrinolytics to enhance vessel patency [7]) and that inhibition of GP IIb-IIIa also has some efficacy in reversing arterial thrombi [8]. Models utilizing intravital microscopy have opened a new era as they permitted real-time analysis of the kinetics of thrombosis and helped discover multiple secondary mediators of thrombus growth and stability (reviewed in [9]). In light of the significant discoveries associated with the use of live microscopy, some laboratories have decided to couple the features of intravital microscopy with perfusion chamber technology to better understand the kinetics of human thrombosis under flow. As a result, it was demonstrated that continuous ADP-P2Y_12_ signaling is critical for maintaining the active conformation level of GP IIb-IIIa to promote thrombus stability [10,11]. This observation, however, required specialized laboratories with expensive best-in-class technology.

Since drugs used to treat arterial thrombosis are believed to affect key mediators of thrombus growth and/or stability we decided to develop a simple perfusion chamber assay that allows for mechanistic studies but also continuous monitoring of the pharmacodynamic activity of antiplatelet agents on the kinetics of thrombosis in real time.

## Results and discussion

The thrombosis profiler (Additional file 1: Figure S1A) consisted of a custom built epifluorescence microscope, a syringe pump and a holding stage (Additional file 1: Figure S1B) in which sits a glass capillary (Vitrocom, 0.2 mm x 2 mm section) coated with human type III fibrillar collagen [12]. In a first set of experiments, we analyzed the homogeneity of the collagen coating. To do so, thrombotic deposits were rinsed for 40 seconds by perfusion of a rinsing buffer, followed by perfusion of a phosphate buffer solution (used for collagen dialysis) supplemented with glutaraldehyde (final concentration of 2.5%). Fixation was continued for another 2 hours. Post-fixation with KMnO_4_, dehydration in ethanol was followed by epon embedding, removal of the glass surface and a second embedding in epon. Evaluation of the thrombotic deposits was performed on semi-thin cross-section cut perpendicular to the direction of the blood flow at the proximal part of the capillary [12]. En Face examination indicated homogenous distribution in size and location of platelet-rich thrombi (Additional file 1: Figure S1C). We next analyzed rhodamine 6 G uptake by platelets in whole blood. Immediately after collection, blood was labeled with rhodamine-6 G (1.25 μg/mL) and maintained at 37°C for the entire course of the experiment. In 10-minute intervals, 5 μL of blood was sampled and diluted into 1.4 mL of sheath fluid buffer (BD, San Jose, CA). Two minutes after the dilution, each sample was analyzed by BD FACSCalibur. The forward scatter (FSC), side scatter (SSC), and fluorescence channel (FL1) were set at logarithmic scale. A total of 10,000 events in the platelet region were collected. The geometric mean fluorescence of platelets was measured with FL1 gain set at 999 (to allow detection of rhodamine 6 G). A total of 5 different samples were analyzed. Data indicated that constant fluorescence of the platelets is achieved after 20 minutes and lasted for at least 70 minutes (Additional file 1: Figure S1D).

### Software development for quantification of platelet deposition

Segmentation, partitioning of an image into non-overlapping regions, was accomplished based on a method proposed by Otsu [13]. This algorithm locates a point in the histogram to minimize the intra-class variance of the foreground and the background. Once the threshold is determined, pixels with values lower than the threshold (e.g., circulating blood) are classified as background and pixels with values greater than the threshold (e.g., platelets and thrombi) are marked as foreground. The success of this thresholding method centers upon whether the proper threshold exists and whether it can be inferred from the image histogram. If, for example, the surface reflectance of the objects to be segmented is not distinct from the background or if the scene is not evenly illuminated then the resulting image histogram would not produce a bimodal or multi-modal graph to allow the computation of best possible threshold. For this reason we adopted a multi-stage segmentation process. Thus after applying the threshold to generate a binary image, morphological operation “closing” (dilation followed by erosion-used to fill in holes and small gaps) followed by morphological operation “opening” (erosion followed by a dilation-used to eliminate all pixels in regions that are too small to contain the structuring element) is applied to join together the thrombi objects and clear the image of small artifacts. Next, a median filter was applied to further reduce the salt-and-pepper noise while simultaneously preserving the edges. The fluorescence intensity of the flowing whole blood that was perfused during the first 20 seconds was used for threshold determination, expressed as background object and not taken into consideration in the calculation of the mean fluorescence intensity. Lastly, a watershed algorithm [14] was applied to identify individual thrombi in the image. Once the image was segmented, the sum of intensity values of pixels inside each foreground object was divided by the corresponding object area (μm^2^) for every frame (sec). Other parameters recorded were the time to reach the peak (sec) and the slope before and after the peak (Additional file 1: Figures S1E, S1F). Similar technology and software analysis can be used for evaluation of the pro-coagulant activity of platelets under flow (data not shown).

### Assay validation

**Figure 1:**
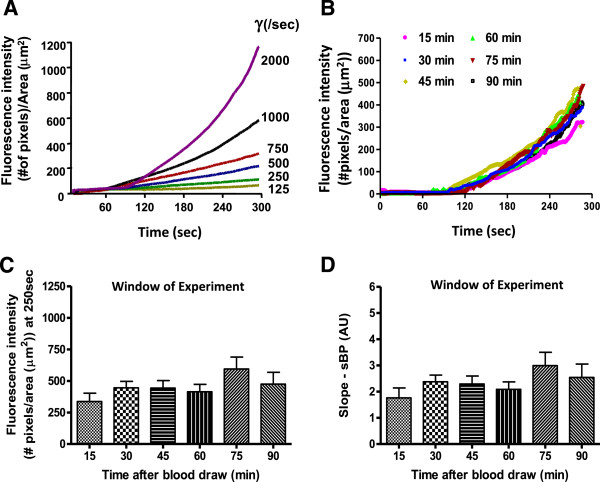
**A)**, **Examples of thrombotic profiles generated by the perfusion of untreated FXa-anticoagulated samples of blood from a healthy individual on fibrillar collagen at different shear rates (representative of 5 different donors).** The profiles lose their linearity as the wall shear rate increases. At least three main factors may contribute to the exponential curve observed for wall shear rates > 500/sec. i) an increase amount of platelets in the vicinity of the collagen surface, ii) a participation of von Willebrand factor in the thrombotic deposits for higher wall shear rate conditions, iii) an exponential increase in shear rate at the apex of the thrombi. As a result, it is expected that exponential profiles could be also observed at lower wall shear rates but for longer perfusion period. **B**), Representative thrombotic profiles of untreated blood perfused at 1000/sec, 15, 30, 45, 60 75 and 90 minutes post blood collection from a single individual. The working range of the assay post blood collection is plotted as mean + S.E.M for Peak thrombus size in **C** and slope before peak in **D.**

**Figure 2:**
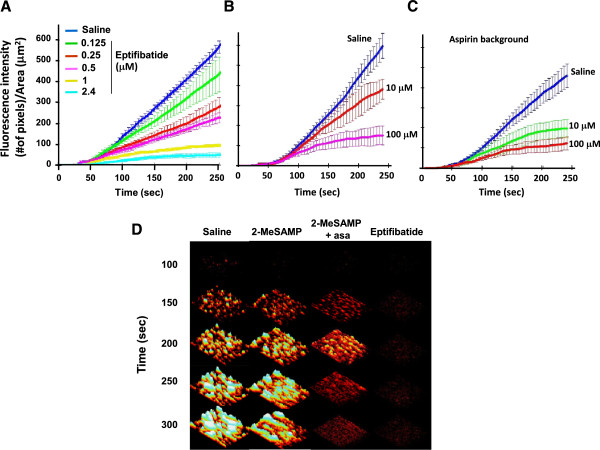
**Representation of the mean ± S.E.M. of the thrombotic profiles of the blood of 7 individuals treated *in vitro *with saline, 2-MeSAMP (10, 100 μM) and eptifibatide (2.4 μM) in absence or presence of aspirin (325 mg/d for 3 days). A**), GP IIb-IIIa antagonism (eptifibatide (μM) spiked *in vitro*) inhibits thrombus growth. A monolayer of platelets is found at the highest dose of eptifibatide (2.4 μM). **B**), Effects of 2-MeSAMP on thrombus formation. **C**), Effects of 2-MeSAMP on thrombus formation on an aspirin background (325 mg/d for 3 days). **D**), representative photomicropgraphs of the thrombotic deposits.

In initial experiments, we established the thrombotic profiles of untreated samples of blood at different wall shear rates (from 125/sec to 2000/sec) by varying the flow rate fixed by the pump (Figure 1A). We then determined the window of experimentation. Six different collagen-coated capillaries were utilized per individual to determine whether the thrombotic profiles (generated at 1000/sec) varied according to the length of time post blood collection (Figure 1B). Data were generated by perfusing whole blood from 6 individuals at 15, 30, 45, 60, 75, and 90 minutes post blood collection. Untreated samples of blood demonstrated identical profiles between T30 and T90 minutes post-blood draw (Table 1; Figure 1B-D; Additional file 2: Video S1). Figure 2C and D demonstrate that neither the maximum peak (C) nor slope before the peak (D) were significantly different at any of the time points measured beyond T15 minutes. The smaller flurorescence detected at the 15 min time-point is likely related to sub-optimal staining (see Additional file 1: Figure S1D). As a result, the window of experimentation was determined to be between 30 and 90 minutes post blood collection.

**Table 1:** **Within donor variability of Peak Fluorescence (Pixels/Total Area (μm**^**2**^**)) and Slope (sBP-Arbitrary Units) of thrombotic profile assessed by RTTP**

**Donor**	**Peak thrombosis (t = 300 sec)**						**Slope (sBP)**					
	**Assay 1**	**Assay 2**	**Assay 3**	**Mean**	**SD**	*CV	**Assay 1**	**Assay 2**	**Assay 3**	**Mean**	**SD**	*CV
1	304	426	346	359	62	0.17	1.08	1.58	1.29	1.32	0.25	0.19
2	887	945	1140	991	132	0.13	3.52	3.45	4.37	3.78	0.51	0.13
3	597	616.	447	554	92	0.17	2.14	2.28	1.73	2.05	0.29	0.14
4	1067	1162	956	1062	103	0.10	4.52	4.62	4.69	4.61	0.08	0.02
5	443	393	463	433	36	0.08	1.89	1.69	1.79	1.79	0.10	0.05
6	904	1024	942	957	61	0.06	3.74	4.59	3.85	4.06	0.47	0.11

The coefficient of variation (CV) was determined by measurement of peak thrombus size (P) and slope of thrombotic process (sBP) for 3 different samples taken from 6 individuals. * CV = (SD/mean).

### Assay reproducibility

Within donor variability was established by perfusing 3 whole blood samples collected on different days from 6 individuals at 1000/sec. Maximum peak and slope were calculated for each sample, outlined in Table 1. Coefficient of variation for each individual was calculated as SD/mean, ranging from 0.06 to 0.17 in maximum peak and from 0.02-0.19 in slope before peak.

### Characterization of the kinetics of thrombosis

Thrombosis triggered by type III collagen followed the classical pattern described by the group of K.S. Sakariassen : i) an increase in thrombus size that parallels that of the wall shear rates (Figure 1A; [15]) an axial dependency as thrombi gradually decreased in size towards the distal part (data not shown). The continuous monitoring revealed a linear increase in thrombus growth for lower wall shear rates (125 to 750/sec), a profile that progressively becomes exponential for higher levels of shear. Similar findings were reported by the group of Dr. McIntire on a collagen type I-vWf-coated surface [16].

### Antithrombotic activities of antiplatelet agents

Eptifibatide, inhibited thrombus growth independently of the shear rates (data not shown), and inhibited thrombus growth in a dose-dependent manner at 1000/sec, reaching a maximal effect at 2.4 μM (Figure 2A).

**Figure 3:**
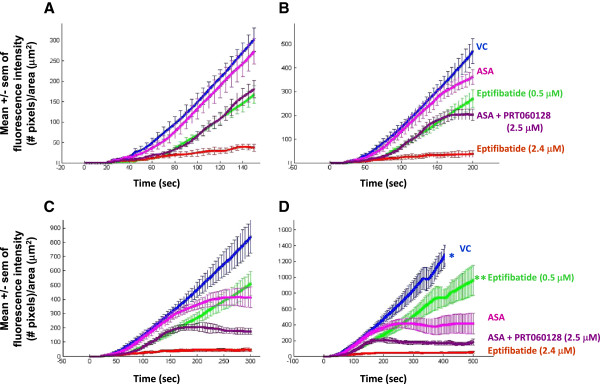
**Representation of the mean ± S.E.M. of the thrombotic profiles of the blood of 5 individuals treated with vehicle control, aspirin (325 mg/day for 3 days), aspirin + PRT060128 (2.5 μM spiked *in vitro *), eptifibatide (0.5 and 2.4 μM) after 150 sec (A), 200 sec (B), 300 sec (C) and 500 sec (D).** Asterisks indicate cessation of blood perfusion following capillary occlusion. Two out of the 5 capillaries occluded in the 0.5 μM eptifibatide group (**).

Previously, we found that genetic targeting of P2Y_12_ promoted a cyclic thrombotic process in injured mesenteric arteries in mice [17]. We investigated how antagonism of the P2Y_12_ receptor would affect human thrombosis. Addition of 2-MeSAMP at 100 μM (a dose that fully inhibits platelet aggregation triggered by 10 μM ADP [12]) did not affect the initial growth of thrombi on collagen but allowed for cyclic thrombotic process characterized by the continuous formation and embolization of small platelet thrombi in the absence of occlusion (Figure 2B; Additional file 3: Video S2). Low antagonism of P2Y_12_ by 2-MeSAMP (10 μM) in presence of aspirin recapitulated the profile obtained with full P2Y_12_ antagonism, consisting in the formation and embolization of platelet-rich thrombi (Figure 2C, D; Table 2 and Additional file 4: Video S3). Since some individuals developed small, while others developed larger thrombi upon pre-treatment with the antiplatelet agents, the time to embolization varies. Accordingly, the mean curve of the different profiles appeared flat. The formation and size of emboli was depicted by the extent of the SEM (for example, see Figure 3D and the curves comparing aspirin alone and aspirin + PRT060128). 

**Table 2:** **Characteristics of the thrombotic profiles generated upon *in vitro *addition of saline, eptifibatide and 2-MeSAMP (2MeS.)**

**Parameters**		**(Saline)**	**2-MeS. (10 μM)**	**2-MeS. (100 μM)**	**Aspirin (asa)**	**2-MeS. (10 μM) + asa**	**2-MeS. (100 μM) + asa**	**Eptifibatide (2.4 μM)**
Maximum Peak (pixels/μm^2^)	Mean Median	596 ± 65 693	309 ± 54 * 318	160 ± 50 ** 109	455 ± 61 423	218 ± 530 **,† 189	127 ± 33 ***, †† 103	63.5 ± 12 *** 53
Time to the peak (sec)	Mean Median	240 ± 0.1 240	216 ± 16 240	197 ± 22 * 231	239 + 0.5 239	214 ± 12 228	204.6 ± 16 *,†† 233	234.4 ± 10 240
Slope before the peak	Mean Median	3.2 ± 0.3 3.1	1.96 ± 0.3 * 1.87	1 ± 0.24 ** 0.94	2.47 + 0.37 2.14	1.33 ± 0.4 **,† 1.13	0.77 ± 0.2 ***,†† 0.43	0.34 ± 0.07 *** 0.3
Slope after the peak	Mean Median	0/7^#^	2/7^#^ −0.3 ± 0.1	4/7^#^ −0.5 ± 0.24	1/7^#^ −0.15	4/7^#^ −1.4 ± 0.66	3/7^#^ −0.07 ± .02	4/7^#^ −0.13 ± 0.05

Compounds were added to untreated or aspirinated blood samples (individuals on a 325 mg/d aspirin dose for 3 days). *, **, *** ; P < 0.05, P < 0.01, P < 0.0001 *vs* baseline, respectively. †, ††, P < 0.05, P < 0.01 *vs* aspirin (n = 7). ^#^ indicates number of curves with reversal.

The importance of capturing the kinetics of thrombosis was further highlighted in Figure 3A-D. Data showed that the antithrombotic activity (both qualitative and quantitative) of the different compounds and their combination varied according to the duration of the blood perfusion and the process (growth or stability) they affect. All capillaries perfused with untreated samples of blood occluded before 500 sec. While aspirin did not show activity at 150 sec, its curve reached a plateau beyond 200 sec. The combination of aspirin with PRT060128 showed stronger antithrombotic activity, reaching an inflexion point after 150 sec. The size of the error bars (sem smaller for the combination of drugs than for aspirin alone) indicated a constant formation and embolization of smaller thrombi (Figure 3C, D). Eptifibatide (0.5 μM, the concentration achieved during the IMPACT-II trial) only affected thrombus growth and could not prevent occlusion in 2 out of the 5 capillaries over the 500 sec period (although the Figure 3A suggested strong activity), indicating ability to delay but not to prevent vascular occlusion. This is in sharp contrast with the interpretation of the Figure 3A that suggested that eptifibatide (0.5 μM) displayed ~ 50% inhibition while aspirin was ineffective. Greater concentration of eptifibatide (2.4 μM) abolished the thrombotic process throughout the entire duration of the experiment (Figure 3D).

### Constant signaling via P2Y_12_ is critical for thrombus stability

**Figure 4:**
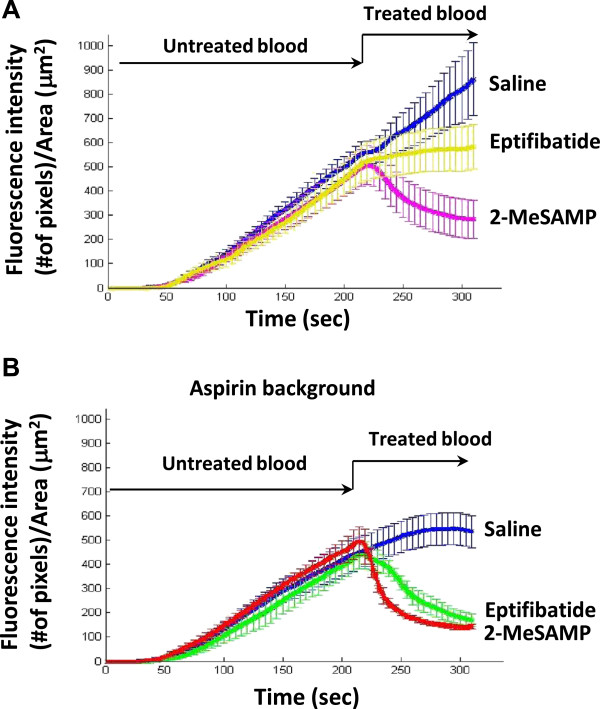
**Effects of 2-MeSAMP and eptifibatide on preformed thrombi.** Representation of the mean ± S.E.M. of the thrombotic profiles generated by perfusion of untreated sample of blood (for 210 sec) immediately followed by an additional perfusion of blood treated with either saline, 2-MeSAMP (100 μM) or eptifibatide (2.4 μM) in (**A**) absence, or (**B**) presence of aspirin.

Since stabilization of arterial thrombi is a function of intraplatelet signaling pathways and is directly affected by the thrombus size and the flow conditions, times to embolization can vary between individuals. By consequence, “flat” thrombotic profiles (see Figure 2B, 3D) are plotted when the mean of multiple experiments is computed and the notion of thrombus stability is represented by the SEM of the curve. To further evaluate the importance of the different receptors (P2Y_12_, GP IIb-IIIa) and enzymes (COX-1) in mediating thrombus stability, we first perfused untreated whole blood samples, then switched to anti-platelet-treated samples of blood. Perfusion of eptifibatide-treated blood blocked further thrombus growth with no apparent destabilization of the preformed thrombi (Figure 4A; mean slope of the treated sample of blood (second perfusion, mSAP: saline, 2.7 ± 0.3 n = 7; eptifibatide, 0.17 ± 0.22, n = 7, p < 0.05 *vs* saline, n = 6). In contrast, addition of 100 μM 2-MeSAMP caused sudden destabilization of preformed thrombi resulting in marked dethrombosis (Figure 4A; mSAP = −1.8 ± 0.2, n = 7, p < 0.001 *vs* eptifibatide; 10 μM, mSAFP = −0.77 + 0.9 (NS)). We also determined the effects of these inhibitors on thrombi formed from blood from aspirin-treated individuals. Aspirin alone (Figure 4B) induced a slow and moderate destabilization of the thrombi (mSAFP = −1.07 ± 0.3). However, perfusion of blood treated with the two inhibitors over preformed thrombi induced dethrombosis (2-MeSAMP (100 μM, mSAP = −4.1 ± 0.7; 10 μM, mSAP = −2.19 ± 0.28); eptifibatide (mSAP = −2.6 ± 0.9); n = 7, p < 0.05 *vs* respective monotherapies and aspirin alone).

### Antithrombotic profiles of clopidogrel, aspirin and their combinations

**Figure 5:**
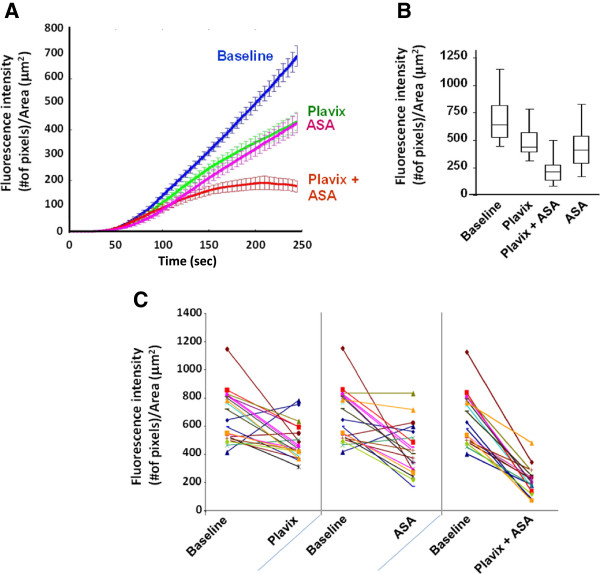
**A)**, **Representation of the mean ± S.E.M. of the thrombotic profiles of twenty individuals successively evaluated at baseline (B.), on clopidogrel (C.), clopidogrel + aspirin (C. + A.), then aspirin (A.) treatments (experiments carried at 1500/sec). B**), Whisker graph depicting the median fluorescence intensity (pixels)/ total area (μm^2^) (on y-axis) at the maximum peak for each treatment condition, showing the maximum and minimum values for n = 20 subjects, as well as 25% and 75% confidence intervals. **C**), Individual variations in fluorescence intensity between baseline and the various treatments.

To determine how aspirin and clopidogrel effected thrombus growth and thrombus stability in this model, we evaluated the effects of chronic dosing of these drugs on the thrombotic profiles of 20 healthy volunteers. These drugs, taken individually or in combination, did not affect the time for the platelets to reach firm adhesion on the collagen surface. Some destabilization of thrombi occurred following clopidogrel and aspirin monotherapies which contributed to an overall decrease in the maximum peak and the slope of the curve (Figure 5A, B and Table 3). Under our experimental conditions, aspirin and clopidogrel showed similar anti-thrombotic activities in normal volunteers with aspirin demonstrating slightly more variable inhibition than clopidogrel. Four out of twenty individuals (blue asterisk) treated with aspirin or clopidogrel did not show significant benefit from the therapy. However, while the combination of clopidogrel and aspirin did not affect the initial growth of the thrombi on collagen (until T 2 minutes), it allowed for marked dethrombosis and cyclic thrombotic process in the twenty donors (Figure 5C).

**Table 3:** Characteristics of the thrombotic profile of healthy subjects

		**Baseline**	**Clopidogrel**	**Clopidogrel + ASA**	**ASA**
Max. Peak (pixels/μm^2^)	M ± sem median	800 ± 51 797	516 ± 48** 526	263 ± 53***,†, ‡ 243	484 ± 67* 460
Time to the peak (sec)	M ± sem Median	240 240	239 ± 1 240	203 ± 12*, † 208	234 ± 6 240
Slope before peak	M ± sem Median	4.3 ± 0.3 4.3	2.7 ± 0.2** 2.7	1.6 ± 0.2***,†, ‡ 1.6	2.7 ± 0.4 2.65
Slope after peak	M ± sem median	NA	1 out of 8# −0.18	5 out of 8# −1.2 ± 0.8	2 out of 8# −0.5 ± 0.4
Thrombus volume (μm^3^/μm^2^)	M ± sem	37 ± 5.2	25.8 ± 3.7	5.4 ± 0.5	22.1 ± 4.8

Healthy subjects were evaluated at baseline, following clopidogrel (75 mg/d for 14 days), clopidogrel (75 mg/d for an additional 7 days) + aspirin (325 mg/d for 7 days), then aspirin (325 mg/day for 14 days) therapies. M (mean), *, **, *** ; P < 0.05, P < 0.01, P < 0.0001 *vs* baseline, respectively. ^†^, P < 0.05 *vs* clopidogrel. ^‡^, P < 0.001 *vs* aspirin. Blood perfusion of 250 seconds; ^#^ indicates number of curves with reversal. Thrombus volume (μm^3^/μm^2^) was evaluated by computer assisted morphometry analysis of cross-sections cut perpendicularly to the blood flow at 8 mm from the proximal end of the capillary.

Perfusion chambers were developed more than 30 years ago to study platelet thrombosis in samples of non-anticoagulated or anticoagulated blood exposed to physiological thrombogenic surfaces under defined conditions of shear (for review, see [18]). Major contributions to the field of thrombosis have been reviewed by Sakariassen and coworkers [19,20]. These include: - the understanding of the mechanisms of inherited clinical disorders associated with platelet pathology (e.g., von Willebrand disease, Glanzmann thrombasthenia, Bernard Soulier Syndrome, etc.…), - the relative role of coagulation factors in thrombosis (Tissue factor, Fibrinogen, FVII, FVIII, FIX, FXI and FXII), - the critical role of platelet membrane glycoproteins (GP IIb-IIIa, GPIb-IX-V and GPVI) in mediating platelet adhesion and thrombus growth under arterial shear rates, - the agonist activities of ADP and TxA_2_. Perfusion chamber technology has also proven instructive in monitoring the antithrombotic activity achieved by antithrombotic therapies and their combination, e.g. clopidogrel plus aspirin. Data showed that the clinical benefits achieved by the combination of these two drugs were superior to each drug used as monotherapy [6,17,21]. Recently published manuscripts by Hosokawa *et al.* measuring perfusion chamber flow pressure changes to evaluate the thrombotic profiles of whole blood support the methodology described here [22,23]. Despite the different quantification methods, the measured effects on thrombus formation and stability were similar- specifically the activities of aspirin alone, P2Y_12_ inhibitor alone, the combination of aspirin and P2Y_12_ inhibitor and the GP IIb-IIIa inhibitor.

As highlighted in this manuscript, three considerations suggest that continuous monitoring of thrombosis at arterial rates of shear would be of value to understand the molecular mechanism regulating arterial thrombosis and to determine the antithrombotic activity achieved in individual patients: First, the assay provides immediate, real time evaluation of the kinetics of thrombosis, it is easy to use, reproducible, detects and differentiates the activity of mono and combination therapies, and can be used as a bedside device. Second, it has become clear from clinical conditions such as unstable angina and from experiments in animals such as those using the Folts model that platelet factors exist that mediate or inhibit thrombus stability. Our data obtained in blood from healthy volunteers confirmed the fact the clinical efficacy of the state-of-the art antiplatelet therapy (clopidogrel + aspirin) utilized in the management of post-atherothrombotic events certainly stems from a synergism in destabilization activities of the two drugs [11,12,24]. They also suggested that high levels of inhibition of the P2Y_12_ receptor may be sufficient to provide substantial benefits in agreement with others [17,25]. The effect of eptifibatide related on the other hand, to the inhibition of thrombus growth. This is particularly important as we found that 0.5 μM eptifibatide (the plasma concentration achieved during the IMPACT-II trial that failed at demonstrating substantial clinical benefits [26]) only delayed the growth of the thrombus, but could not prevent occlusion of the collagen-coated capillary upon prolonged perfusion (Figure 3). Third, emerging data suggest that the antithrombotic response to aspirin and clopidogrel is variable, possibly including non-responders [27,28]. Three out of 20 healthy individuals did not benefit from either aspirin or clopidogrel treatments, suggesting non-responsiveness. Interestingly however, all individuals showed robust inhibition while on combination regimen, indicating that the mechanisms targeted (COX-1 or P2Y_12_) while inhibited, could not account for antithrombotic activity without concomitant inhibition of the other signaling pathway.

### Limitations

While this methodology for the evaluation of anti-platelet therapies has obvious benefits, there are also limitations that warrant discussion: i) use of a single purified protein surface rather than damaged endothelial cells- the choice of collagen can affect the thrombotic process therefore careful standardization of the collagen preparation (or protein of choice) is required ii) use of anticoagulant- a method for evaluation in absence of anticoagulant at low and high shear would facilitate the study of existing therapies on both fibrin formation and platelet deposition simultaneously. Based on the optimized conditions used in this study (high shear, and Factor Xa anticoagulant), our results relate to platelet rich thrombi only.

## Conclusions

Coupling the real time feature of intravital technology with perfusion chamber has been proposed by the group of McIntire almost 20 years ago [4]. This concept was based on the simple idea that real time monitoring of perfusion chambers would circumvent the limitations of light transmittance aggregometry and other point of care devices which either: i) do not offer continuous monitoring of the kinetics of arterial thrombosis, ii) interrogate only one platelet signaling pathway at a time and therefore cannot provide reliable monitoring of combination therapies. The system described herein offers a relatively simple and reproducible technology that can be utilized to: i) characterize the mechanism of action and activity of antiplatelet or anticoagulant agents ii) possibly tailor patient therapy [29], iii) identify novel antiplatelet targets.

## Methods

### Real time perfusion chamber assay

Blood was collected from the antecubital vein of subjects who gave written informed consent to the protocol (Approval was obtained from Western Institutional Review Board or the California Pacific Medical Centre Human Subjects Committee) via butterfly 19 G on syringes containing 5 μM (final concentration) of the factor Xa inhibitor C921-78, a concentration sufficient to block any FXa activity [30].

Platelets were labeled in whole blood by adding an aliquot of Rhodamine 6 G (final concentration, 1.25 μg/ml). To visualize platelet deposition through the microscope, the dye was excited with light from a high-power light emitting diode with a spectral maximum at 530 nm and a spectral half width of 35 nm (Luxeon V, Lumileds Lighting, San Jose, CA). Excitation and emission lights were filtered with a set of fluorescence filters (31002, Chroma Technologies, Rockingham, VT). A microscope objective imaged an area of 360 x 270 μm on the internal wall of the capillary onto a Sony XCD X-710 digital camera (resulting magnification ca. 13 x). Images were recorded at a frequency of 1 Hz. Blood flow was established by a syringe pump withdrawing blood through the capillary (Harvard Apparatus, Holliston, MA). A personal computer with custom software was used to control the camera and the syringe pump, and to display and record images and experimental conditions.

Unless otherwise specified, whole blood was perfused through the capillary at 1000 or 1500/sec for a period of 5 minutes according to the following equation: γ = 1.03x6Q/ab^2^, where γ = shear rate; Q = volumetric flow rate (ml/60); b = height (cm); a width (cm).

### Antithrombotic activities of antiplatelet agents

Eptifibatide (added in *vitro* at 0.125 to 2.4 μM) and 2-MeSAMP (a direct P2Y_12_ antagonist added *in vitro* at 10 and 100 μM, a concentration that fully antagonizes P2Y_12_[12]) effects were tested in absence or presence of aspirin (325 mg QD for 3 days) at 1000/sec. Qualitative and quantitative changes in thrombotic profiles of 5 individuals treated with vehicle control, aspirin, aspirin + PRT060128 (2.5 μM, [31]) or eptifibatide (0.5 and 2.4 μM) were also assessed over a 500 second perfusion period.

In a second set of experiments, we asked whether addition of inhibitors of the receptors for fibrinogen (GP IIb-IIIa) or ADP (P2Y_12_) to preformed thrombi could induce dethrombosis. Untreated blood samples were perfused at 1500/sec through the chamber for 210 seconds, followed by blood treated with saline, 2.4 μM eptifibatide, or 2-MeSAMP (10, 100 μM) for 100 seconds. Similar design was applied to the blood of normal volunteers who had been taking aspirin (325 mg/d) for 3 consecutive days.

### Monitoring the antithrombotic activities of clopidogrel, aspirin and clopidogrel + aspirin in humans

Antithrombotic effects were evaluated in a sequential study at the California Pacific Medical Centre (CPMC, San Francisco, CA). Twenty individuals were evaluated before antithrombotic therapy, 2 weeks after a daily regimen of clopidogrel (75 mg/d), 1 week after a dual clopidogrel (75 mg/d) + aspirin (325 mg/d) therapy, and finally 2 weeks off clopidogrel therapy while continuing on aspirin regimen (325 mg/d).

### Statistical analysis

Statistics were performed with Graph Pad Prism v4.03 and numerical data are presented as mean ± S.E.M. The significance between two data sets was tested using paired t-test for the CPMC study, and unpaired t-test for all other in vitro studies. *p* < *0.05* was considered to be statistically significant*.*

## Competing interests

All authors are present employees of Portola Pharmaceuticals and/or former employees of Millennium Pharmaceuticals.

## Authors contributions

GS and PA designed and executed experiments. GS and PA wrote the manuscript. MH, CW, MJ conducted experiments. H-CL, GS, KM, and CM designed, executed hardware and software of the assay. PBC and DRP contributed to scientific design of the studies. All authors read and approved the final manuscript.

## Supplementary Material

Additional file 1 **Figure S1. **A), Illustration of the real time thrombosis profiler. (1), Harvard Apparatus mono-syringe pump; (2), thermostat controller; (3), on/off switches of the pump, lamp source and thermostatic holder; (4–6), control knobs for 3-axis sample translation stage; (7), holding stage; (8), 20X Nikon microscope objective; The Sony XCD-X710 camera is mounted on top of the objective (not shown) B), Holding stage. The glass capillary is placed between 2 sets of springs, the holding stage slide on two steel bars to its position under the objective of the microscope. This reduces the need for X, Y, and Z focusing. C), Left, en face representative picture of thrombotic deposits formed at the proximal part of the capillary. Thrombi were rinsed, fixed and stained with toluidine blue following perfusion of untreated whole blood (1000/sec, 5 minutes perfusion) through the collagen coated perfusion chamber as previously described. Right, semithin cross-section of epon-embedded thrombotic deposits cut perpendicular to the direction of the blood flow at the proximal part of the capillary. D), Time course of the labeling of platelets with rhodamine 6 G as determined by FACS analysis. T0 corresponds to endogenous fluorescence intensity of the platelets prior to incubation with R6G. E), Computer. F), Schematic of the parameters recorded for each curve, maximum peak (P), time to reach the peak (T), slope before the peak (sBP), and slope after the peak (sAP). Different antithrombotic profiles are presented as an example. Click here for file

Additional file 2 **Video S1. **Three-dimensional reconstruction of the thrombotic process (untreated sample of blood) triggered by type III collagen at 1500/sec. Relative increase in platelet accumulation is indicated by progression in color from red to white peaks. Click here for file

Additional file 3 **Video S2. **Full P2Y_12_ antagonism induced dethrombosis. Click here for file

Additional file 4 **Video S3. **Thrombotic process triggered by type III collagen at 1500/sec in an individual treated with aspirin (325 mg/d for 3 days and 10 µM 2-MeSAMP). Click here for file
