# Supplementary figures and images for: Development of a perfusion chamber assay to study in real time the kinetics of thrombosis and the antithrombotic characteristics of antiplatelet drugs
Source: Thromb J. 2012 Aug 1;10:11. doi: 10.1186/1477-9560-10-11 (PMC3502277; doi:10.1186/1477-9560-10-11)

## Slide 1
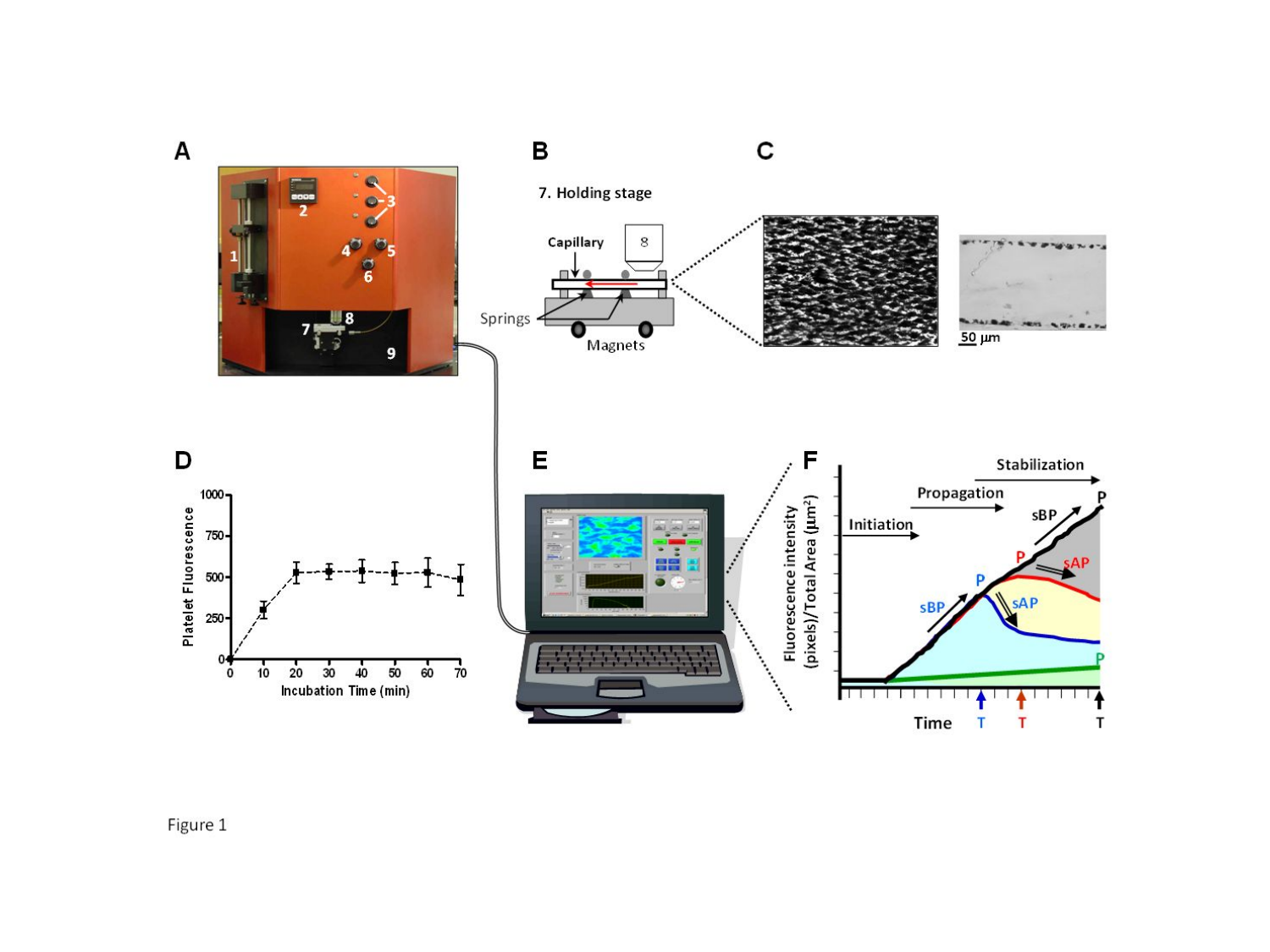

Supplement: Additional file 1 — Figure S1. A), Illustration of the real time thrombosis profiler. (1), Harvard Apparatus mono-syringe pump; (2), thermostat controller; (3), on/off switches of the pump, lamp source and thermostatic holder; (4–6), control knobs for 3-axis sample translation stage; (7), holding stage; (8), 20X Nikon microscope objective; The Sony XCD-X710 camera is mounted on top of the objective (not shown) B), Holding stage. The glass capillary is placed between 2 sets of springs, the holding stage slide on two steel bars to its position under the objective of the microscope. This reduces the need for X, Y, and Z focusing. C), Left, en face representative picture of thrombotic deposits formed at the proximal part of the capillary. Thrombi were rinsed, fixed and stained with toluidine blue following perfusion of untreated whole blood (1000/sec, 5 minutes perfusion) through the collagen coated perfusion chamber as previously described. Right, semithin cross-section of epon-embedded thrombotic deposits cut perpendicular to the direction of the blood flow at the proximal part of the capillary. D), Time course of the labeling of platelets with rhodamine 6 G as determined by FACS analysis. T0 corresponds to endogenous fluorescence intensity of the platelets prior to incubation with R6G. E), Computer. F), Schematic of the parameters recorded for each curve, maximum peak (P), time to reach the peak (T), slope before the peak (sBP), and slope after the peak (sAP). Different antithrombotic profiles are presented as an example. [file 1477-9560-10-11-S1.ppt]
